# Late Diagnosis and Entry to Care after Diagnosis of Human Immunodeficiency Virus Infection: A Country Comparison

**DOI:** 10.1371/journal.pone.0077763

**Published:** 2013-11-05

**Authors:** H. Irene Hall, Jessica Halverson, David P. Wilson, Barbara Suligoi, Mercedes Diez, Stéphane Le Vu, Tian Tang, Ann McDonald, Laura Camoni, Caroline Semaille, Chris Archibald

**Affiliations:** 1 Centers for Disease Control and Prevention, Atlanta, Georgia, United States of America; 2 Public Health Agency of Canada/Agence de la santé publique du Canada, Ontario, Canada; 3 Kirby Institute, University of New South Wales, Sydney, New South Wales, Australia; 4 Istituto Superiore di Sanità, Rome, Italy; 5 Secretaría del Plan Nacional sobre el Sida/Centro Nacional de Epidemiología, Madrid, Spain; 6 Institut de Veille Sanitaire, Saint –Maurice, France; 7 ICF Marcro International, Atlanta, Georgia, United States of America; The Hospital for Sick Children and The University of Toronto, Canada

## Abstract

**Background:**

Testing for HIV infection and entry to care are the first steps in the continuum of care that benefit individual health and may reduce onward transmission of HIV. We determined the percentage of people with HIV who were diagnosed late and the percentage linked into care overall and by demographic and risk characteristics by country.

**Methods:**

Data were analyzed from national HIV surveillance systems. Six countries, where available, provided data on two late diagnosis indicators (AIDS diagnosis within 3 months of HIV diagnosis, and AIDS diagnosis within 12 months before HIV diagnosis) and linkage to care (≥1 CD4 or viral load test result within 3 months of HIV diagnosis) for people diagnosed with HIV in 2009 or 2010 (most recent year data were available).

**Principal Findings:**

The percentage of people presenting with late stage disease at HIV diagnosis varied by country, overall with a range from 28.7% (United States) to 8.8% (Canada), and by transmission categories. The percentage of people diagnosed with AIDS who had their initial HIV diagnosis within 12 months before AIDS diagnosis varied little among countries, except the percentages were somewhat lower in Spain and the United States. Overall, the majority of people diagnosed with HIV were linked to HIV care within 3 months of diagnosis (more than 70%), but varied by age and transmission category.

**Conclusions:**

Differences in patterns of late presentation at HIV diagnosis among countries may reflect differences in screening practices by providers, public health agencies, and people with HIV. The percentage of people who received assessments of immune status and viral load within 3 months of diagnosis was generally high.

## Introduction

Diagnosis of human immunodeficiency virus (HIV) infection soon after acquisition and prompt entry into care are part of national strategies in most high-income countries for minimizing ongoing HIV transmission. People who present with advanced HIV disease have higher morbidity and mortality than those diagnosed early and can unknowingly transmit HIV for a longer period of time [1], [2]. HIV-infected people who engage in care soon after initial HIV diagnosis have a lower risk for premature mortality and are more likely to achieve viral suppression and lower viral load burden [3], [4]. People who do not engage in care may have relative poor treatment adherence [5], [6], resulting in reduced viral suppression and increased secondary transmission [7]–[12]. Early initiation of care is also essential to provide opportunities for additional activities for HIV prevention through health care, including screening and counseling for risk behaviors [13].

In high-income countries, about a quarter to a third of people with HIV are diagnosed late, with a CD4+ T-lymphocyte count of <200 cells/µL or an illness that defines the acquired immunodeficiency syndrome (AIDS). However, this may vary by country and by population subgroup. In Europe, overall about 29% of people are diagnosed with late stage HIV disease [14]; with a range of 23–45% of people diagnosed late across countries [15], [16]. Estimates from other high-income countries are 20% in Australia [17], 28% in the United States [18], 17% in British Columbia, Canada [19], 30%–37% in Spain [20], [21], 30% in France [22], and 28–39% in Alberta, Canada [23], [24]. Late presentation differs by risk group, generally with a lower percentage diagnosed late among men who have sex with men (MSM) than people who inject drugs (IDU) or heterosexuals [16]–[18], [21], [22], [25]–[27], and by age, with higher percentages presenting late among older age groups [15], [18], [21], [26], [27].

The percentage of people diagnosed with HIV who are linked to care may also vary by country and population subgroups. However, little population-based information is available on linkage to care, which is generally defined as evidence of having at least one CD4+ T-lymphocyte count or viral load test result within 3 months of HIV diagnosis. In the United States, an estimated 77% of people diagnosed with HIV are linked to care; however, this varies by race/ethnicity, age, and transmission category [18], [28]. A study in Ontario, Canada, found that 64.1% of people were linked to care within 3 months following diagnosis, and 75.2% were linked to care within 12 months [29]. A study in Northern Alberta, Canada, found that 92% of individuals diagnosed with HIV infection accessed care following their diagnosis; 70% within 2 months of diagnosis [24]. Both Canadian studies also found that time to care varied by race/ethnicity, age, exposure category, and geography.

Differences in late diagnosis with HIV disease or linkage to care between countries may be due to differences in individual and system level factors such as health care delivery and access to care. Differences may also result from lack of uniformity in definitions for late diagnosis or linkage to care, or from data based on particular subpopulations. We analyzed data from the national HIV surveillance systems of Australia, Canada, France, Italy, Spain, and the United States and used uniform definitions to determine the percentage of people with HIV who were diagnosed late and the percentage linked into care overall and by demographic and risk characteristics.

## Methods

HIV surveillance data can be used to monitor stage of disease at HIV diagnosis and care visits by examining CD4 count and viral load data for HIV-infected people. We used data from HIV surveillance systems in Australia, Canada, France, Italy, Spain, and the United States to determine the percentage of people diagnosed late with HIV and who were linked to care after HIV diagnosis as measured by CD4 count and viral load (VL) tests overall and by demographic and risk characteristics. The countries included in the analyses agreed to participate among member countries from Europe, North America, and Australia that participate in an informal annual meeting to discuss HIV surveillance. Each country maintains a national surveillance system with country-specific reporting rules and representation of the population.

National HIV surveillance is coordinated in Australia by the Kirby Institute, in collaboration with the State and Territory health authorities, the Australian Government Department of Health and Ageing, and other public health surveillance networks [30]. Diagnoses are notifiable to state and territory health authorities and have been recorded for AIDS since 1981 and HIV since 1985. State and territory health authorities forward information about newly diagnosed cases of HIV infection including demographic and clinical characteristics, and self-report of exposure to HIV, to the Kirby Institute for entry into the National HIV Registry [31]. In Canada, all thirteen provinces and territories voluntarily submit non-nominal information on new HIV diagnoses to the Public Health Agency of Canada, including some demographic information and reported risk factors [32]. AIDS diagnoses and other clinical information are not uniformly collected and reported across all jurisdictions, and different data sub-sets were created for each variable to capture cases only from jurisdictions where reporting of that variable was systematic and consistent to avoid the potential bias of underreporting. Thus, Canadian data presented here may not be nationally representative. In France, HIV case reporting was implemented in 2003 in addition to AIDS case reporting. HIV case reporting to the Institut de Veille Sanitaire (InVS), the French Institute for Public Health Surveillance, is mandatory and anonymous; anonymous codes are created by microbiologists and clinicians provide epidemiological and clinical data, including clinical stage at the time of HIV diagnosis [33]. CD4 count was introduced in data collection in 2008 and cannot be used yet to assess access into care. In Italy, the reporting of HIV cases became mandatory in 2008. Data are reported to the National Institute of Health since 2008 from region and provinces. Data are included from all regions except Sardegna (coverage 97.8%); in 2010, data on CD4 were available from 18 out of 21 regions and provinces (coverage 87.5%). In Spain, all autonomous regions collect information on new HIV and AIDS diagnoses and send them to the National Centre of Epidemiology in Madrid, but countrywide coverage for new HIV diagnoses has been achieved only in 2012. Case definitions follow the European guidelines. Demographic, risk factors and clinical data are collected in each case, including CD4 count at first determination after diagnosis; however, information on viral load is not collected and the date of CD4 determination is only available in 7 autonomous regions. In the United States, all states and U.S. dependent areas report information on people diagnosed with HIV infection to the Centers for Disease Control and Prevention’s (CDC) National HIV Surveillance System, including demographic information, risk factors, and clinical information, including AIDS diagnoses. However, as of now, complete data on CD4 and viral load test results has been reported from only 14 areas.

Generally, late diagnosis has been defined as a CD4+ T-lymphocyte count of <200 cells/µL [23], [26] or a combination of a CD4 value of <200 cells/µL or an opportunistic illness [18]. Other definitions have been proposed, such as a definition of very late diagnosis for people with CD4<200 cells/µL and late diagnosis with CD4<350 cells/µL based on treatment recommendations in 2010 [1], [34]. In the United States, recent guidelines recommend all people with HIV be offered treatment [35], [36]. For this analysis, we defined late diagnosis as a CD4<200 cells/µL or presence of an opportunistic illness within 3 months of diagnosis among people newly diagnosed with HIV. We also assessed late diagnosis with a second definition because not all countries had data available to meet the first definition: percentage of people who had an HIV diagnosis within 12 months before AIDS diagnosis, among people diagnosed with AIDS in 2010. Entry to care was defined as ≥1 CD4 or viral load test within 3 months of diagnosis; in Spain only data on CD4 test were used since information on viral load test within 3 months of diagnosis is not available.

We determined late diagnosis and linkage to care overall and by age, sex, and transmission category. Analyses were conducted by the respective country HIV surveillance program and data were provided where available and for the most recent year available (2009 or 2010).

## Results

### Late Diagnosis (CD4<200 Cells/µL or Presence of an Opportunistic Illness within 3 Months of Diagnosis) among People Newly Diagnosed with HIV (Table 1)

Information on late diagnosis was available from Australia, Canada, France, Italy, and the United States. In each country, the majority of diagnoses of HIV infection were among men and among people aged 20–49 years old (Table 1). In Australia, Canada and the United States, the largest number of people diagnosed with HIV reported their exposure to HIV as male-to-male sexual contact; while in France and Italy a higher number of infections was attributed to heterosexual contact. The highest percentage of people diagnosed with late stage disease was among people in the United States (28.7%), followed by Australia (18.8%), France (15.2%), Italy (14.5%), and Canada (8.8%). Late diagnosis was generally higher among older age groups. Late diagnosis was either similar for males and females (Italy) or higher among males (Australia, Canada, France, United States). The pattern of late diagnosis by transmission category differed between countries: compared to MSM, the percentage diagnosed late was higher for people with infection attributed to heterosexual contact in Australia, France, and Italy (absolute difference ≥5%), and among IDU in France and the United States.

**Table 1:** Number of people diagnosed with HIV infection and percentage with CD4<200 or diagnosed with opportunistic illness at or within 3 months of HIV diagnosis, by country of residence.

	Australia^a^		Canada^b^		France^c^		Italy^d^		United States^e^	
	Total	Diagnosed with AIDS within 3 months of HIV diagnosis	Total	Diagnosed with AIDS within 3 months of HIV diagnosis	Total	Diagnosed with AIDS within 3 months of HIV diagnosis	Total	Diagnosed with AIDS within 3 months of HIV diagnosis	Total	Diagnosed with AIDS within 3 months of HIV diagnosis
	N	%	N	%	N	%	N	%	N	%
Total	1051	18.8	1472	8.8	6265	15.3	3839	14.5	43130	28.7
Age, years										
0–9	6	0.0	5	0.0	41	17.1	12	0.0	179	7.3
10–19	16	6.3	19	0.0	162	6.8	34	2.9	2110	12.8
20–29	267	9.0	342	2.6	1441	7.6	772	6.9	13179	16.8
30–39	340	17.1	456	6.8	2035	13.1	1309	11.5	10427	29.9
40–49	261	24.1	399	13.8	1480	18.4	1074	17.5	9995	37.3
50 or older	161	32.3	248	13.7	1107	26.3	630	25.9	7240	42.1
Unknown			3.0	0.0			8	12.5		
Sex										
Male	896	18.4	1165	9.5	4199	16.5	2889	14.3	33609	29.1
Female	150	21.3	306	5.9	2066	12.8	943	15.3	9521	27.4
Transgender	5	20.0					7	0.0		
Transmission category^f^										
MSM	655	13.3	257	7.4	2459	11.8	1165	11.5	25892	26.7
IDU	23	26.1	92	8.7	74	27.0	254	16.1	3470	39.1
MSM-IDU	22	27.3	13	7.7					1329	28.0
Heterosexual	278	29.5	231	8.7	3645	17.4	1796	18.1	12184	30.4
Other	73	23.3	12	16.7	88	14.8	142	2.8	256	22.1
Unknown							482	11.0		

a Includes cases of HIV infection, newly diagnosed in Australia in 2010 and reported by 31 March 2012. The exposure category “Heterosexual contact” includes cases from high prevalence countries in sub-Saharan Africa and specific countries in South East Asia (Burma, Cambodia).

b Dataset is not nationally representative; it includes data from 5 of the 13 provinces and territories. Across all 13 provinces and territories, a total of 2,358 HIV cases were reported in 2010. The case definition for AIDS in Canada is based on confirmed HIV diagnosis and presence/diagnosis of an AIDS-defining condition (no criteria for CD4 count are included in Canada’s AIDS case definition).

c Data for the whole country, adjusted for under-reporting and reporting delays. Missing data on age,sex, transmission category and clinical stage are imputed. Data reported as of 30 June, 2011. MSM-IDU are in “others”.

d In 2010 the coverage of HIV surveillance system is 97.8%. AIDS is undereported. AIDS defined as clinical stage C (CDC classification).

e Includes cases of HIV infection, newly diagnosed in the United States in 2010 and reported by 31 December 2011. Estimated numbers resulted from statistical adjustment that accounted for missing risk-factor information, but not for reporting delays and incomplete reporting.

f MSM, men who have sex with men; IDU, injection drug use.

### Late Diagnosis (HIV Diagnosis within 12 Months before AIDS Diagnosis) among People with AIDS (Tables 2 and 3)

Data were available from all 6 countries on the percentage of people diagnosed with HIV within 12 months of AIDS diagnosis (Tables 2 and 3). The majority (53–65%) of people diagnosed with AIDS were diagnosed with HIV in the 12 months prior to their AIDS diagnosis. There were no clear trends by age and compared to females, males had higher percentages diagnosed within 12 months in all countries except Australia. Compared to MSM, a higher percentage of people diagnosed with AIDS whose infection was attributed to heterosexual contact had their first HIV diagnosis in the 12 months prior in Australia, Canada, France, and Italy and percentages were lower among IDU compared to MSM in all 6 countries.

**Table 2:** Percentage of people who had an HIV diagnosis within 12 months before AIDS diagnosis, among people diagnosed with AIDS in 2010, by country of residence–Australia, Canada, and France.

	Australia^a^		Canada^b^		France^c^	
	Diagnosed with AIDS, total	Diagnosed with HIV within 12 months before AIDS diagnosis	Diagnosed with AIDS, total	Diagnosed with HIV within 12 months before AIDS diagnosis	Diagnosed with AIDS, total	Diagnosed with HIV within 12 months before AIDS diagnosis
	N	%	N	%	N	%
Total	123	65.0	247	64.0	1613	64.8
Age, years						
0–9	0		1	0.0	5	100.0
10–19	1	100.0	1	0.0	9	100.0
20–29	12	66.7	24	45.8	169	74.2
30–39	38	65.8	64	60.9	449	62.9
40–49	37	59.5	88	70.5	523	58.0
50 or older	35	68.6	69	66.7	458	69.9
Sex						
Male	112	64.3	201	67.7	1,113	68.09
Female	11	72.7	46	47.8	500	57.41
Transmission category^d^						
MSM	67	59.7	32	62.5	477	64
IDU	3	33.3	59	42.4	111	28
MSM-IDU	3	0.0	9	11.1	2	0
Heterosexual	39	82.1	33	66.7	1,001	70
Other	11	63.6	4	75.0	21	42

a New diagnoses of AIDS in Australia, reported by 31 March 2012. AIDS notifications are accepted as being an incomplete record of AIDS diagnoses in Australia in 2010.

b This dataset is not nationally representative; it includes data from 7 of the 13 provinces and territories. The case definition for AIDS in Canada is based on confirmed HIV diagnosis and presence/diagnosis of an AIDS-defining condition (no criteria for CD4 count are included in Canada’s AIDS case definition). Transmission category information was not available for one of the jurisdictions reported here, which accounts for the small cell sizes under the various transmission categories.

c Data for the whole country, adjusted for under-reporting and reporting delays. Data reported as of 30 June, 2011.

d MSM, men who have sex with men; IDU, injection drug use.

**Table 3:** Percentage of people who had an HIV diagnosis within 12 months before AIDS diagnosis, among people diagnosed with AIDS in 2010, by country of residence–Italy, Spain, and United States.

	Italy^a^		Spain^b^		United States^c^	
	Diagnosed with AIDS, total	Diagnosed with HIV within 12 months before AIDS diagnosis	Diagnosed with AIDS, total	Diagnosed with HIV within 12 months before AIDS diagnosis	Diagnosed with AIDS, total	Diagnosed with HIV within 12 months before AIDS diagnosis
	N	%	N	%	N	%
Total	1102	62.9	930	56.7	26,599	53.3
Age, years						
0–9	2	100.0	2	100.0	14	92.9
10–19	4	75.0	1	100.0	481	67.8
20–29	97	72.2	100	72.0	4,636	58.3
30–39	309	69.9	274	55.8	6,831	52.5
40–49	429	52.9	367	48.5	8,320	50.3
50 or older	261	67.0	186	65.1	6,317	53.5
Sex						
Male	816	64.8	720	58.6	19,902	56.2
Female	286	57.3	210	50	6,697	44.8
Transmission category^d^						
MSM	247	67.2	246	71.5	13,474	58.7
IDU	213	27.7	263	18.6	3,494	43.1
MSM-IDU	4	75.0			1,223	36.4
Heterosexual	537	73.6	308	71.4	8,173	52.3
Other	101	69.3	6	66.7	236	24.4
Missing			107	72.9		

a National AIDS Register include the whole country. Data collected at 12/31/2011.

b Spanish AIDS Register which covers the whole country. Information on date of of HIV dianosis is available in 900 (96.8%) of AIDS cases notified to the Register.

c New diagnoses of AIDS, reported by 31 December 2011.Estimated numbers resulted from statistical adjustment that accounted for missing risk-factor information, but not for reporting delays and incomplete reporting.

d MSM, men who have sex with men; IDU, injection drug use.

### Linkage to Care (Table 4)

Data were available on linkage to care from Australia, Canada, Italy, Spain, and the United States (Table 4). Overall linkage was high, with 89.9% linked to care in Australia, 89.6% in Italy, 80.3% in the United States, 76.0% in Spain, and 72.6% in Canada. Linkage to care was lower among adolescents and younger adults compared to older adults in Australia, Canada, and the United States. Compared to MSM, a greater percentage of IDU were linked to care in Australia; while fewer were linked in Spain, Italy, and Canada. The percentage linked to care was lower among people with HIV infection attributed to heterosexual contact in Australia and Canada, compared to MSM.

**Table 4:** Number of people diagnosed with HIV infection and percentage who had a CD4 and/or viral load test within 3 months of HIV diagnosis, by country of residence.

	Australia^a^		Canada^b^		Italy^c^		Spain^d^		United States^e^	
	Total	≥1 CD4 or VL within 3 months of diagnosis	Total	≥1 CD4 or VL within 3 months of diagnosis	Total	≥1 CD4 or VL within 3 months of diagnosis	Total	≥1 CD4 or VL within 3 months of diagnosis	Total	≥1 CD4 or VL within 3 months of diagnosis
	N	%	N	%	N	%	N	%	N	%
Total	1054	89.8	976	72.6	3245	89.6	1519	76.0	6674	80.3
										
Age, years										
0–9	6	83.3	5	80.0	8	87.5	7	57.1		
10–19	16	81.3	14	71.4	26	84.6	20	85.0	344	74.7
20–29	269	87.7	208	69.2	620	90.6	424	74.5	2134	76.4
30–39	340	89.7	314	71.3	1083	91.0	557	75.4	1577	80.0
40–49	262	91.2	262	74.0	946	89.4	357	77.3	1570	85.2
50 or older	161	91.9	172	77.3	554	87.2	154	78.6	1049	82.8
Missing			1	0	8	25.0				
Sex										
Male	898	90.2	725	74.2	2451	89.5	1273	77.0	5267	79.8
Female	151	89.4	236	67.8	787	90.5	246	70.7	1407	82.1
Transgender	5	20.0			7	28.6				
Transmission category^f^										
MSM	656	90.9	231	72.7	1012	92.1	765	81.6	4109	79.8
IDU	23	100.0	66	63.6	236	83.1	67	64.2	527	78.7
MSM-IDU	22	90.9	8	100.0					238	83.4
Heterosexual	280	85.0	208	68.3	1506	91.7	487	76.8	1792	81.3
Other	73	74.0	44	68.2	140	83.6	9	66.7	7	94.7
Missing							191	56.0		

a Cases of HIV infection newly diagnosed HIV infection in Australia in 2010 including cases previously diagnosed overseas. All new diagnoses inAustralia in 2010, reported by 31 March 2012. All new diagnoses includes cases previously diagnosed overseas, some of whom have received treatment for HIV infection. The heterosexual contact category includes 53M, 80F, total 133 cases from high prevalence countries and 42M, 12F, total 54 cases whose exposure was attributed to heterosexual contact with a partner from a high prevalence country.

b This dataset is not nationally representative; it includes data from 4 of the 13 provinces and territories and one of these jurisdictions was incomplete (nominal cases only). Across all 13 P/Ts, a total of 2,358 HIV cases were reported in 2010.

c HIV surveillance includes the whole country except Sardegna region (coverage 97.8%). Data reported by 31 December 2010.

d Data Source: New HIV Diagnoses Information System (SINIVIH in Spanish). In 2011, SINIVIH was implemented in 17 out of 19 Autonomous Region, and coverage was 71% of the total Spanish population. While information on first CD4 count after diagnosis is available in all Regions, information on DATE of CD4 count determination is currently available only in seven (Aragon, Asturias, Canary Islands, Castile-Leon, Madrid, Murcia and Navarre). This seven regions provided 1519 (52.2%) of the total 2907 new HIV diagnoses notified in 2010. Information on date of CD4 count was missing in 248 (16%) of the 1519 new HIV diagnoses. Information on viral load determination within 3 months after HIV diagnosis is not collected.

e Includes cases of HIV infection diagnosed in 14 jurisdictions of the United States and reported by 31 December 2011. Estimated numbers resulted from statistical adjustment that accounted for missing risk-factor information, but not for reporting delays and incomplete reporting. Age group 10–19 includes 13–19 only.

f MSM, men who have sex with men; IDU, injection drug use.

## Discussion

The percentage of people presenting with late stage disease at HIV diagnosis varied by country, overall with a range from 28.7% (United States) to 8.8% (Canada), and by transmission categories. The percentage of people diagnosed late was lower in several countries than previously reported (e.g., Canada) [24], [23]. The percentage of people diagnosed with AIDS who had their initial HIV diagnosis within 12 months before AIDS diagnosis varied little among countries, except the percentages were somewhat lower in Spain and the United States. Overall, the majority of people diagnosed with HIV were linked to HIV care within 3 months of diagnosis (more than 70%), but varied by age and transmission category. Improvements in detecting HIV earlier and differences in patterns of late presentation at HIV diagnosis among countries may reflect differences in screening practices by providers, public health agencies, and people with HIV.

Screening recommendations, screening patterns independent of screening recommendations, and access to health care influence early detection of HIV infection. Some countries have shifted from HIV testing based on risk assessment to HIV screening in certain settings. For example, in 2006 the U.S. CDC recommended routine HIV screening in clinical settings where HIV prevalence is >0.1 percent and expanded support to state health departments for HIV testing [37]. However, the percentage diagnosed late was higher in the United States compared to the other countries included in this analysis. Another factor that can impact access to care is lack of health insurance, with 32% of Latinos, 21% of blacks, and 16% of whites being uninsured in the United States [38]. Generally, MSM test more frequently and have lower percentages of late presentation. The higher percentage presenting late for people with infection attributed to heterosexual contact in Australia, France, and Italy may be related to lack of perceived risk or a denial of risk and therefore failure to test for HIV, or a higher percentage of foreign-born people. The higher percentage presenting late among IDU in the United States may be due to not accessing health care due to lack of insurance (United States), social disadvantage, lack of social/familial support, or stigma associated with drug use behaviors. The situation is different in France compared with the United States. Most people who use intravenous drugs in France have been infected with HIV in the 1990s and most have been diagnosed many years ago [39]. Very few people who use intravenous drugs were newly diagnosed with HIV, that is less than 100 cases were diagnosed during the analysis year, representing less than 1% of all new HIV cases, and half of these cases were immigrants. Moreover, the second indicator used in this analysis (HIV diagnosis within 12 months before AIDS) shows that only 28% of IDU in France were diagnosed with HIV within 12 months before AIDS diagnosis, a low percentage compared to other categories (70% among heterosexuals, 64% among MSM).

Similar factors may affect linkage to care among IDU (e.g., in Canada and Spain, fewer IDU were linked to care compared with MSM). Effective drug treatment programs may include HIV prevention services, such as HIV testing and linkage to care. Similar to earlier findings from North America [22], [28], [29], in most countries a lower percentages of younger people was linked to care soon after diagnosis compared to older people. Younger people may feel healthy and therefore may not feel the need to engage in care soon after diagnosis. In general, people diagnosed with HIV should receive an initial assessment of immune system status and viral load promptly after diagnosis [35], [36], and with guidelines now recommending treatment be offered to people with less severe disease (e.g., WHO guidelines recommend treatment at CD4 count ≤350 cells/mm^3^) [40] prompt linkage to care could improve worldwide.

Because not all countries could provide data on stage of disease at diagnosis, we also assessed AIDS diagnosis in relation to the time of HIV diagnosis (Tables 2 and 3). This measure reflects people with AIDS who may have been recently diagnosed or people who were diagnosed with HIV years before their AIDS diagnosis and who may or may not have been on treatment. While this measure is not directly comparable to the late diagnosis measure presented in Table 1, and does not reflect people who may have developed AIDS but were promptly treated, it can indicate a need for earlier detection and prompt treatment.

Our analyses are subject to several limitations. Reporting of HIV diagnoses and HIV-related laboratory test results to HIV surveillance differ between countries. For some countries information was not available for the entire population. Classification of severe stage of HIV (i.e., AIDS) may also differ between countries, depending on whether only CD4 test results are considered and/or opportunistic illnesses. We assumed that people who did not have a CD4 count did not have AIDS since if they had severe disease, they would have been diagnosed with AIDS. Our results need to be interpreted in light of the treatment guidelines and health care systems of the respective countries. We measured linkage to care based on CD4 and viral load test results; we did not have information on clinic visits that may not have resulted in such testing. Incomplete reporting of test results may have underestimated linkage to care.

In summary, the percentage with advanced disease at HIV diagnosis and linkage to care vary between the high-income countries included in this analysis. In general, MSM are diagnosed earlier than people with infection attributed to other risk factors. The percentage of people who received assessments of immune status and viral load within 3 months of diagnosis was generally high. Future analyses may need to explore factors within countries that promote higher benchmarks on these indicators and support early diagnosis and prompt linkage to care.
